# Onion peel tea ameliorates obesity and affects blood parameters in a mouse model of high-fat-diet-induced obesity

**DOI:** 10.3892/etm.2013.1433

**Published:** 2013-11-28

**Authors:** SHOGO MATSUNAGA, KAZUO AZUMA, MAYUMI WATANABE, TAKESHI TSUKA, TOMOHIRO IMAGAWA, TOMOHIRO OSAKI, YOSHIHARU OKAMOTO

**Affiliations:** 1 Faculty of Agriculture, Tottori University, Tottori 680-8553, Japan; 2 Finarl Co., Ltd., Tottori 680-1167, Japan

**Keywords:** onion peel, tea, anti-obesity, leptin, functional food

## Abstract

The present study examined the effects of onion peel tea (OPT) in a mouse model of high-fat-diet-induced obesity. BALB/c mice were fed a high-fat diet for three weeks, followed by a normal diet with or without OPT for 28 days. OPT suppressed the increases in body weight and level of epididymal fat tissue; it also significantly reduced the serum concentrations of total cholesterol on day 14 and those of glucose and leptin on day 28. The results indicate that OPT has anti-obesity effects in an experimental mouse model of high-fat-diet-induced obesity.

## Introduction

Obesity is an increasing global health problem, and has been associated with metabolic syndrome (MetS), diabetes, cardiovascular disease, hypertension and cancer (1). The increasing incidence of obesity suggests that this epidemic is likely to worsen in the future (2). Animal models are useful tools for evaluating the potential efficacy of compounds for the prevention and treatment of obesity. It has been demonstrated that rodents fed a high-fat diet are good models of obesity, in which the dietary environment is a major contributor (3).

It has been previously shown that certain foods are beneficial for the suppression or prevention of MetS, including tea (4). Green tea is already a popular beverage, and may be easily incorporated as part of a diet designed to attenuate or prevent the symptoms of MetS (4). Catechins, in particular, are one of the major polyphenolic compounds in tea and are beneficial for the treatment of the main MetS conditions, including obesity and type-2 diabetes, as well as cardiovascular risk factors (5). Another potentially beneficial food is onion. Onion has the capacity to regulate lipid metabolism and suppress hyperglycemia and diabetes (6). Previous studies have attributed the anti-obesity effects of onion to quercetin, one of the flavonoids present in onion peel (7–9). Tea extracted from onion peel (onion peel tea; OPT) may thus be expected to exert beneficial effects in MetS.

In the present study, the anti-obesity effects of OPT were evaluated in mice that had been fed a high-fat diet. Furthermore, the effects of OPT on blood parameters in these mice were examined.

## Materials and methods

### OPT

The present study used freeze-dried OPT, containing 1.15 mg/g quercetin. The OPT (Sarratto tamacha) was acquired from Finarl Co., Ltd. (Tottori, Japan).

### Animals and diets

Twenty BALB/c mice (male, 4 weeks old) were purchased from CLEA Japan, Inc. (Osaka, Japan). The animals were maintained under standard conditions. The use of these animals and the procedures they were subjected to were approved by the Animal Research Committee of Tottori University (Tottori, Japan). Throughout the experimental period, the mice had unrestricted access to food and water.

### Study design

The mice were randomized into the control and OPT groups (n=10 mice per group). Following habituation, all mice were fed a high-fat diet (HFD: High Fat Diet-32; CLEA Japan, Inc.) from day −21 to day 0. The control group was then fed a normal powdered diet (CE-2; CLEA Japan, Inc.) from day 0 to day 28, while the OPT group was fed a normal powdered diet supplemented with 5% (w/w) OPT. The mice were weighed once every seven days from day −21 to day 28.

Blood and epididymal fat tissue were harvested from the mice on days 14 and 28 (n=5 at each time-point). Blood was collected via cardiac puncture under isoflurane inhalation anesthesia. After allowing to stand for 1 h at room temperature, the serum was recovered by centrifugation of the blood at 1,000 × g for 10 min at 4°C. The serum samples were then stored at −80°C prior to analysis. Following blood collection, the animals were immediately sacrificed by cervical dislocation, and their epididymal fat tissue was harvested and weighed.

### Blood chemical analysis

Blood chemicals were measured using a blood chemical auto analyzer (Dry-Chem 7000; Fujifilm Inc., Tokyo, Japan). Serum triglyceride (TG), total-cholesterol (T-cho), glucose (Glu), alanine transaminase (ALT), aspartate aminotransferase (AST), alkaline phosphatase (ALP), geranylgeranyltransferase (GGT) and albumin (Alb) levels were measured.

### Measurement of serum leptin concentration

A sandwich enzyme-linked immunosorbent assay kit (Morinaga Institute of Biological Science, Inc., Yokohama, Japan) was used to measure the leptin concentration, in accordance with the manufacturer’s instructions.

### Statistical analysis

Statistical analyses were performed using the Student’s t-test or one-way analysis of variance (ANOVA) and the Tukey-Kramer test. The data are presented as the mean ± standard deviation. P<0.05 was considered to indicate a statistically significant difference.

## Results

### Effects of dietary OPT on body weight and epididymal fat tissue

**Figure 1:**
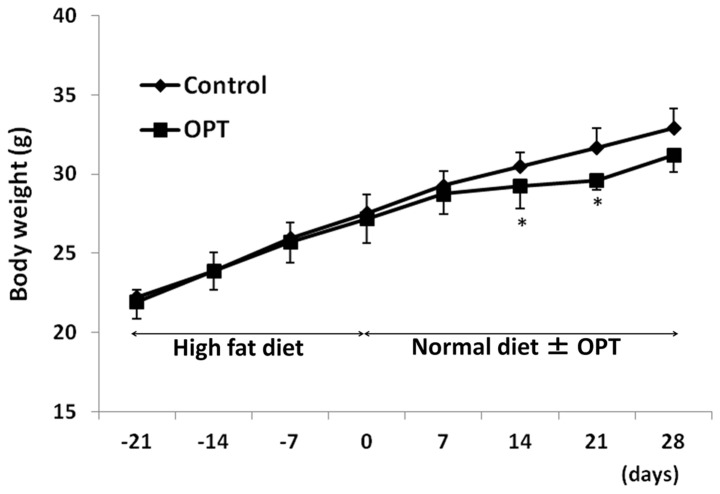
Effects of onion peel tea (OPT) on body weight in a high-fat-diet-induced model of obesity. The data points shown represent the means ± standard deviation; n=5. ^*^P<0.05 compared with the control group, as assessed using the Student’s t-test.

In the OPT group, the intake of quercetin during the experimental period was calculated to be 5–6 mg/kg/day. During the experimental period, the body weights of the mice were observed to increase in the control and OPT groups (Fig. 1). However, in the OPT group, the gain in body weight was attenuated compared with that of the control group. On days 14 and 21, in particular, the body weights in the OPT group were significantly lower than those of the control group (P<0.05).

**Figure 2:**
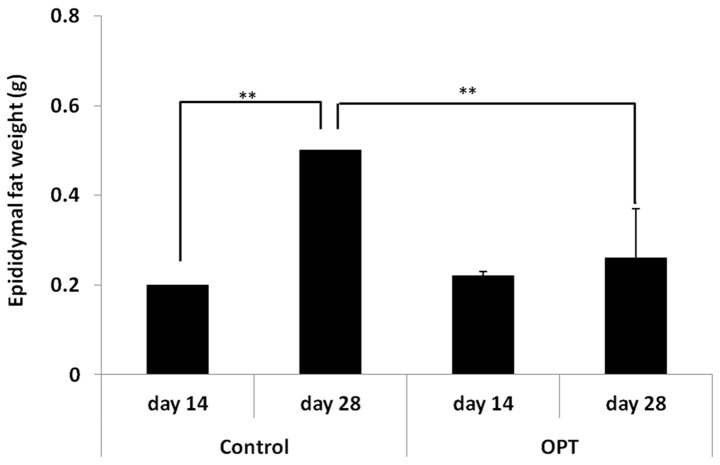
Effects of onion peel tea (OPT) on the weight of epididymal fat tissue in a high-fat-diet-induced model of obesity. The data shown represent the means ± standard deviation; n=5. ^**^P<0.01 as determined with the Tukey-Kramer test.

The mean epididymal fat tissue weights are shown in Fig. 2. On day 14, no significant difference was identified between the epididymal fat tissue weights in the control group (0.2±0.0 g) and the OPT group (0.2±0.1 g). However, on day 28, the mean epididymal fat tissue weight of the OPT group (0.3±0.1 g) was significantly lower than that of the control group (0.5±0.0 g) (P<0.01).

### Effects of OPT on blood chemical parameters

The blood chemical analysis results are shown in Table I. On day 28, the mean serum Glu concentration in the OPT group was significantly lower than that of the control group (P<0.01). On day 14, however, there was no significant difference in serum Glu concentration between the groups. On day 14, the mean serum concentration of T-cho in the OPT group was significantly lower than that of the control group (P<0.05). On day 28, however, there was no significant difference in mean serum T-cho concentration between the groups. On day 28, the mean serum ALP concentration in the OPT group was significantly higher than that of the control group (P<0.01), although there was no difference on day 14. In the OPT group, the mean serum TG concentrations were lower than those of the control group on days 14 and 28, although these differences were not statistically significant. No statistically significant differences were identified between the groups with regard to the serum concentrations of ALT, AST, GGT or Alb on days 14 or 28.

**Figure 3:**
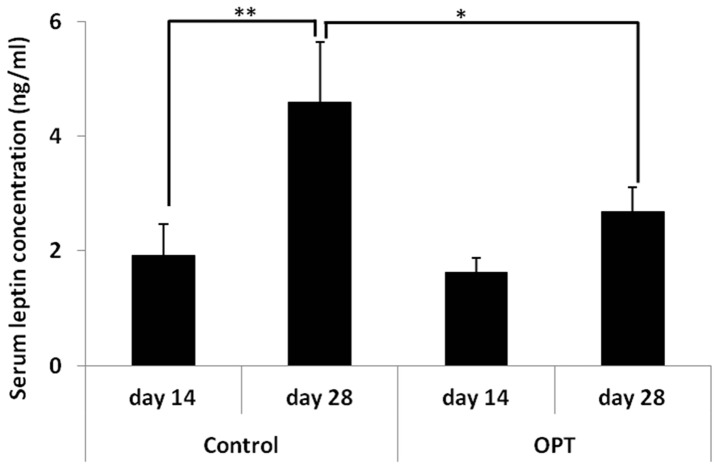
Effect of onion peel tea (OPT) on serum leptin concentration in a high-fat-diet-induced model of obesity. The data shown represent the means ± standard deviation; n=5. ^*^P<0.05, ^**^P<0.01 as determined with the Tukey-Kramer test.

The serum leptin levels of the mice are shown in Fig. 3. On day 14, no significant difference was observed in the leptin level between the control group (1.9±0.5 ng/ml) and the OPT group (1.6±0.2 ng/ml). The mean serum leptin concentration in the control group was significantly higher on day 28 than that on day 14 (P<0.01). Furthermore, on day 28, the mean serum leptin concentration in the OPT group (2.7±0.4 ng/ml) was significantly lower than that of the control group (4.9±1.0 ng/ml) (P<0.05).

## Discussion

In this study, dietary OPT was shown to suppress the increases in body weight and level of epididymal fat tissue in an experimental mouse model. It has been revealed that, in rodents, a high-fat diet is a major contributor to obesity (3). The results of the present study indicated that dietary OPT was capable of reducing body weight in an experimental high-fat-diet-induced model of obesity.

It has previously been suggested that dietary supplementation with onion may be beneficial for improving lipid metabolism in humans; Lee *et al*(10,11) demonstrated that onion peel extract (OPE) was rich in quercetin and was capable of altering the expression of genes involved in cholesterol metabolism. It has also been demonstrated that OPE is able to lower blood low-density lipoprotein cholesterol and increase high-density lipoprotein cholesterol levels by increasing the expression of low-density lipoprotein receptor mRNA and mRNA encoding ATP-binding cassette transporter A1 genes (12). Moreover, a review by Sudathip *et al*(4) reported that green tea extract decreased blood Glu, TG and T-cho levels in diet-induced models of obesity (4). The results of the present study indicated that OPT also suppressed increases in blood Glu, TG and T-cho in a diet-induced model of obesity. Marques *et al*(13) demonstrated that the blood ALP levels were increased in a diet-induced mouse model of obesity. To the best of our knowledge, the correlation between obesity and an increase in blood ALP has not been completely elucidated. Further studies are required in this respect.

It has previously been indicated that the adipocytes in adipose tissue secrete a variety of proteins, known as adipocytokines, including tumor necrosis factor-α, interleukin-6, resistin, leptin and adiponectin (14). Plasma leptin concentrations are positively correlated with adiposity (excessive body fat) and body weight changes in humans and rodents (15). Adiponectin contributes to insulin sensitivity and fatty acid oxidation, and circulating concentrations of adiponectin are inversely correlated with body mass (16,17). The results of the present study indicated that OPT suppressed the secretion of leptin from adipocytes. The suppression of leptin secretion may have contributed to the reduction in body weight and the weight of epididymal fat tissue observed in the mice in the OPT group.

A high intake of plant foods (i.e. vegetables, legumes and fruits), which contain flavonoids and polyphenolic compounds, has been directly correlated with the management and prevention of obesity, type-2 diabetes mellitus and other cardiovascular disease risk factors; among these plant foods, onions (*Allium cepa*) are one of the richest sources of flavonoids in the human diet (18–20). Quercetin is a major flavonol that is abundant in plant products, particularly in onions, and it has been demonstrated to possess antioxidative, anti-inflammatory and lipid-regulating properties (18,21–24). Numerous studies involving human clinical investigations, animal trials and *in vitro* experiments have demonstrated that phenolic substances, including quercetin, have important anti-inflammatory and anti-obesity properties (23,25,26). OPT is rich in quercetin (1.15 mg/g). One possible mechanism by which OPT exerts anti-obesity effects may be via the action of quercetin. In the previous studies, the experimental animals were fed more quercetin than was taken in by the animals in the present study (9,12). The results of the present study suggest that there may be another mechanism underlying the action of OPT. To enhance the understanding of any such additional mechanisms, an analysis of all of the components of OPT may be required.

In conclusion, OPT suppressed the increases in body weight and epididymal fat tissue weight normally observed with a high-fat-diet-induced model of obesity. It also significantly reduced the serum levels of T-cho and Glu. Furthermore, in the OPT group, the level of serum leptin on day 28 was significantly reduced compared with that of the control group. In combination, the results of the present study indicate that OPT may be a potent functional food for the treatment, management or prevention of obesity.

## Figures and Tables

**Table I:** Effects of OPT on blood chemical parameters in a high-fat-diet-induced model of obesity.

	Control		OPT	
		
Parameter	Day 14	Day 28	Day 14	Day 28
Glu (mg/dl)	172.6±17.4	304.2±19.6	185.6±15.2	242.4±28.0^a^
T-cho (mg/dl)	87.4±10.5	97.0±11.1	73.4±5.6^b^	99.0±13.0
TG (mg/dl)	116.0±30.2	186.8±8.1	79.8±9.5	134.4±45.0
ALT (IU/l)	34.0±4.3	35.4±9.9	36.0±6.6	35.6±13.1
AST (IU/l)	100.8±58.6	54.6±13.8	73.6±15.9	50.4±11.0
ALP (IU/l)	385.2±44.4	366.8±12.0	412.0±99.3	422.6±11.0^a^
GGT (IU/l)	6.6±2.2	4.4±0.5	5.4±0.5	4.4±0.5
Alb (g/dl)	2.1±0.1	2.3±0.1	2.1±0.1	2.2±0.1

Data are presented as the mean ± standard deviation; n=5.

a P<0.01,

b P<0.05 compared with the control group, as assessed using the Tukey-Kramer test.

OPT, onion peel tea; Glu, glucose; T-cho, total cholesterol; TG, triglyceride; ALT, alanine transaminase; AST, aspartate aminotransferase; ALP, alkaline phosphatase; GGT, geranylgeranyltransferase; Alb, albumin.
